# Genetic variants in *AKR1B10* associate with human eating behavior

**DOI:** 10.1186/s12863-015-0189-9

**Published:** 2015-03-25

**Authors:** Kerstin Rohde, Martin Federbusch, Annette Horstmann, Maria Keller, Arno Villringer, Michael Stumvoll, Anke Tönjes, Peter Kovacs, Yvonne Böttcher

**Affiliations:** IFB AdiposityDiseases, University of Leipzig, Leipzig, Germany; Max-Planck-Institute for Human Cognitive and Brain Sciences, Department of Neurology, Leipzig, Germany; Department of Medicine, University of Leipzig, Leipzig, Germany; Clinic of Cognitive Neurology, University of Leipzig, Leipzig, Germany

**Keywords:** Eating behavior, Association, Restraint, Disinhibition, Genetic variants

## Abstract

**Background:**

The human *Aldoketoreductase 1B10* gene (*AKR1B10*) encodes one of the enzymes belonging to the family of aldoketoreductases and may be involved in detoxification of nutrients during digestion. Further, *AKR1B10* mRNA (messenger ribonucleic acid) expression was diminished in brain regions potentially involved in the regulation of eating behavior in rats which are more sensitive to cocaine and alcohol. We hypothesized that the human *AKR1B10* gene may also play a role in the regulation of human eating behavior.

**Results:**

We investigated the effects of 5 genetic variants of *AKR1B10* on human eating behavior among 548 subjects from a German self-contained population, the Sorbs, and in 350 subjects from another independent German cohort. Among the Sorbs, we observed nominal associations with disinhibition at the 5′ untranslated region (5′ UTR) variant rs10232478 and the intragenic variants rs1834150 and rs782881 (all *P* ≤ 0.05). Further, we detected a relationship of rs1834150 and rs782881 with waist, smoking consumption (rs782881) and coffee consumption (rs1834150) (all *P* ≤ 0.05). Albeit non-significant, replication analyses revealed similar effect directions for disinhibition at rs1834150 (combined *P* = 0.0096). Moreover, in the replication cohort we found rs1834150 related to increased restraint scores with a similar direction as in the Sorbs (combined *P* = 0.0072).

**Conclusion:**

Our data suggest that genetic variants in the *AKR1B10* locus may influence human eating behavior.

**Electronic supplementary material:**

The online version of this article (doi:10.1186/s12863-015-0189-9) contains supplementary material, which is available to authorized users.

## Background

The human aldoketoreductase 1B10 (AKR1B10) was identified in human hepatocellular cancer as a member of the AKR superfamily [1]. The NADP(H)-dependent (nicotinamide adenine dinucleotide phosphate) enzyme is mostly expressed in the gastrointestinal tract, particularly in the small intestine, and colon as well as adrenal gland [1,2]. It reduces a wide spectrum of natural or synthetic harmful components present in daily nutrients and may hence be involved in detoxification mechanisms [3]. AKR1B10 is further involved in fatty acid biosynthesis [4,5] as well as the prostaglandin and retinoid acid metabolism [6-8]. It was shown that up-regulation of the mRNA or protein expression results in a tumorigenic cell proliferation [7] in several types of cancers such as liver [9], lung [10] and breast cancer [11]. Interestingly, its up-regulation was triggered by smoking in healthy individuals [10,12] or in cells treated with several toxic chemical compounds such as the antioxidant ethoxiquin or lipid derived aldehydes like HNE (Hydroxynonenal) [13], which are also present in daily food. This suggests protective effects of AKR1B10 against toxic nutrient-specific aldehydes [3,14]. In addition, *Akr1b10* expression was shown to be lower in brain of Lewis rats (LEW) that are more sensitive to cocaine or ethanol compared to a control group of rats (Fischer 344 (F344)) [15]. Higueras-Matas et al. (2011) [15] observed a decreased expression of *Akr1b10* in nucleus accumbens and the frontal cortex, areas suggested to be involved in eating behavior. Taken together, (i) the important role of AKR1B10 in detoxifying aldehydes and several other deleterious compounds during digestion and (ii) its putative central effects in brain regions potentially involved in eating behavior allowed us to hypothesize that *AKR1B10* may play a role in human eating behavior.

There is a good evidence for the role of genetics in human eating behavior [16-19] or in altered eating behavior e.g. eating disorders [20]. For instance, risk variants of the *fat mass and obesity associated* gene *(FTO*) were not only associated with higher body mass index (BMI) but also with loss of control over eating and selection of food higher in calories and fat [21-23]. Single nucleotide polymorphisms (SNP) in the *glutamate decarboxylase* gene (*GAD2*) were significantly associated with increased disinhibition and hunger in women from the Quebec Family Study [24] while a promoter SNP was related to disinhibition and hunger scores in morbidly obese patients from a French cohort [25]. Meyre et al. (2005) further described the promoter variant in *GAD2* associated with lower birth weight and subsequently higher BMI Z-Scores in French children [26]. Further, genetic variants within the *leptin* gene (*LEP*) and *cholecystokinin* gene (*CCK*) were shown to be associated with “snacking behavior” [27] and bigger meal sizes [28]. Dotson and colleagues (2010 & 2008) [16,29] found that the density of bitter taste receptors is partially determined by genetic factors influencing eating behavior through sensitivity to bitterness. In the present study we investigated SNP markers within the *AKR1B10* gene and in the upstream untranslated regulatory region. We evaluated its genetic association with human eating behavior, a variety of metabolic and anthropometric phenotypes, and consumption of consumer goods such as alcohol intake, smoking behavior and coffee consumption. To the best of our knowledge this is the first study investigating *AKR1B10* variants and their potential relationship with anthropometric and metabolic parameters as well as eating behavior traits.

## Methods

### Subjects

#### Sorbs

In the present study we used a self-contained population, the Sorbs from Germany, comprising 1046 individuals as described earlier [30] (The entire recruitment was done by Dr. Anke Tönjes and Dr. Jan Halbritter (University of Leipzig, Germany). Sampling was carried out population based. Exclusion criteria were age under 18 years and pregnancy. No specific inclusion criteria in regard to disease states were considered. For subjects recruitment, the study was advertised by flyers, newspapers, radio and by local general practitioners. 618 Sorbs out of 1046 completed the German version of the three factor eating questionnaire (FEV) which determines the three main eating behavior factors - disinhibition, hunger and restraint as described in detail elsewhere [18,31,32]. In the here presented study type 2 diabetes (T2D) was the only exclusion criterion because diabetic individuals usually control their food intake according to specific nutrition plans. Therefore, 70 Subjects with T2D have been excluded from the study. Diabetes was diagnosed based on a 75 g oral glucose tolerance test (OGTT) and the definition of T2D according to ADA criteria [33]. Finally, the study included 548 subjects (346 women and 202 men). No further exclusion criteria were used in this study. All individuals underwent extensive phenotyping [30,34] including a standardized interview for past medical history, family history, measurements of anthropometric parameters (waist, hip, BMI, waist to hip ratio (WHR)), measurements of glucose and insulin metabolism (75 g OGTT, blood glucose and insulin levels at several time points (0, 30 and 120 min after meal) [35]) and measurements of lipid metabolism (total cholesterol levels, high and low density lipoprotein cholesterol levels (HDL-C and LDL-C), and triglyceride concentration (TG)) [36]. Insulin was measured with the Auto-DELFIA Insulin assay (PerkinElmer Life and Analytical Sciences, Turku, Finland). Serum glucose was measured by the hexokinase method (Automated analyser Modular, Roche Diagnostics, Mannheim, Germany). Total serum cholesterol and TG concentrations were measured by standard enzymatic methods (CHOD-PAP and GPO-PAP; Roche Diagnostics). Serum HDL and LDL cholesterol concentrations were determined with commercial homogeneous direct measurement methods (Roche Diagnostics). All assays were performed in an automated clinical chemistry analyzer (Hitachi/ Roche Diagnostics) at the Institute of Laboratory Medicine, University Hospital Leipzig. The main characteristics of the participants are summarized in Table 1.

**Table 1:** **Main characteristics of the study populations**

	**Sorbs**	**Replication**
***N*** **total**	548	350
Age (years)	48 ± 16	27 ± 5
BMI (kg/m^2^)	26.1 ± 4.4	27.0 ± 6.2
Waist-Hip Ratio	0.86 ± 0.1	n.a.
Waist circumference	88.39 ± 13.02	n.a.
Hip circumference	102.65 ± 8.09	n.a.
Fasting plasma glucose (mmol/l)	5.24 ± 0.49	n.a.
30 min plasma glucose (mmol/l)	8.36 ± 1.65	n.a.
120 min plasme glucose (mmol/l)	5.41 ± 1.66	n.a.
Fasting plasma insulin (pmol/l)	37.74 ± 22.71	n.a.
30 min plasma insulin (pmol/l)	290.32 ± 173.40	n.a.
120 plasma glucose (pmol/l)	173.40 ± 156.88	n.a.
Total cholesterol (mmol/l)	5.34 ± 1.08	n.a.
HDL-cholsterol (mmol/l)	1.69 ± 0.41	n.a.
LDL-cholesterol (mmol/l)	3.37 ± 0.99	n.a.
Triglycerides (mmol/l)	1.23 ± 0.83	n.a.
Disinhibition	4.3 ± 3.0	6.2 ± 3.2
Hunger	3.9 ± 2.9	5.5 ± 3.4
Restraint	7.9 ± 4.8	6.1 ± 4.4
Coffee intake (cups per day)	2 ± 1	n.a
Alcohol intake (glasses per week)	3 ± 1	n.a.
Smoking behavior (cigarettes per day)	10 ± 8	n.a.

Data are presented as mean ± standard deviation (SD) BMI: Body Mass Index; WHR: waist-to-hip ratio. Women/men per population is as follows: Sorbs (346 women, 202 men); Replication (152 women, 198 men) n.a.: not available.

### Consumption of consumer goods in sorbs

Consumption of consumer goods such as coffee and alcohol consumption as well as smoking behavior were assessed by standardized interviews for all subjects (no previously validated questionnaire was used). To estimate coffee and alcohol intake, subjects were asked for average intake but not for a certain time period (alcohol: 1) none; 2) occasionally at special events; 3) 2–3 glasses of wine or bottle of beer per week; 4) 1 glass of wine or bottle of beer per day; 5) 2 glasses of wine or bottles of beer per day; 6) more than 3 glasses of wine or bottles of beer per day; coffee: number of cups per day) [30,37]. We did not account for different contents of alcohol per glass/bottle or caffeine content per cup. Smoking behavior was obtained by asking for the time period of smoking (currently, former, never) and the number of cigarettes per day. It was not accounted for occasional smokers.

### Replication cohort

For replication purposes we used another independent German cohort as described elsewhere [18] including 350 healthy individuals (152 women and 198 men). Briefly, phenotyping included anthropometric measurements (BMI and weight), age, sex and data for eating behavior, which were assessed using the German version of the three factor eating questionnaire (FEV) [31]. The main characteristics of the subjects are summarized in Table 1.

All human studies were approved by the ethics committee of the University of Leipzig and all subjects gave written informed consent.

### Genotyping

Five *AKR1B10* SNPs (rs1834150; rs782881; rs3778828; rs4732036; UTR SNP rs10232478) were selected for the analysis. We selected tagging SNPs for our analysis which are in linkage disequilibrium (LD) with several other SNPs within and around the *AKR1B10* locus to enlarge the probability to find associating variants in the gene without the need to analyze all existing SNPs. The genotype information for rs10232478 was extracted from genome wide Affymetrix SNP data [38] for the Sorbs and individually genotyped in the replication cohort. The remaining variants were taken from the HapMap database (r^2^ > 0.8, minor allele frequency (MAF) < 0.05) and genotyped using the KASPar SNP genotyping system (KBiosiences Ltd., Hoddesdon, United Kingdom) according to manufacturer’s protocol (TD PCR (touch down polymerase chain reaction) with following conditions: 94°C for 15 sec, 94°C for 20 sec, 61°C for 60 sec, for 10 cycles, drop down 0.6°C per cycle, 94°C for 10 sec, 55°C for 60 sec, for 26 cycles). Genomic deoxyribonucleic acid (DNA) for genotyping was extracted from blood with QIAmp DNA Blood Midi Kit (Qiagen, Hilden, Germany) according to the manufacturer’s protocol. Fluorescence was detected using ABI PRISM 7500 Sequence Detecting System. To guarantee genotyping reproducibility, a random ~5% of the samples were re-genotyped in all SNPs; all genotypes matched initial designated genotypes. The average genotyping success rate for all analyzed SNPs was 96.38%.

### Statistical analysis

Statistical analyses were performed using the SPSS statistics software version 20.0 (SPSS, Inc.; Chicago, IL). Non-normally distributed parameters were log_10_-transformed to approximate normal distribution prior to analysis. All variants were in Hardy-Weinberg-Equilibrium. Genetic association with eating behavior traits, anthropometric and metabolic phenotypes were calculated using linear regression models adjusted for age, gender and lnBMI (except for BMI). Cronbach’s alpha coefficients were calculated to measure the internal reliability of the three eating behavior subscales. Coffee consumption was defined as cups per day, smoking behavior as number of cigarettes per day and alcohol intake as number of glasses per week. We did not account for different contents of alcohol per glass/bottle or caffeine content per cup. *P*-values for smoking, alcohol intake and coffee consumption were corrected for age and gender. Additive, dominant and recessive models of inheritance were tested. To correct for multiple testing we lowered the significance threshold to (0.05/(315 tests) = 1.6×10^−4^). All *P*-values > 1.6×10^−4^ but ≤ 0.05 were considered to be of nominal statistical significance. All *P*-values are provided uncorrected for multiple testing. Meta-analyses were performed using METAL [39]. All analyses were standardized to the minor allele (plus strand).

## Results

### Association analysis in sorbs

We observed nominally significant associations with disinhibition at the variant rs10232478 located in the 5′ untranslated region and the intronic SNPs rs1834150 and rs782881 (Table 2). For each of the variants the minor alleles were related to increased disinhibition scores. A trend for nominal association with restraint scores was shown at rs3778828. No relationships were obtained for any of the variants with hunger (Table 2).

**Table 2:** **Association analysis of** ***AKR1B10*** **variants in the Sorbs population**

***AKR1B10*** **genotype**															
	**rs10232478**			**rs782881**			**rs1834150**			**rs3778828**			**rs4732036**		
**Genotype (** ***N*** **)**	**CC (76)**	**CT (230)**	**TT (195)**	**CC (114)**	**AC (263)**	**AA (150)**	**TT (115)**	**TA (257)**	**AA (171)**	**AA (37)**	**GA (201)**	**GG (293)**	**CC (49)**	**TC (210)**	**TT (280)**
**Association analysis with anthropometric traits**															
BMI (kg/m^2^)	25.96 ± 3.83	26.42 ± 4.94	25.99 ± 4.09	26.5 ± 4.9	25.9 ± 4.4	26.2 ± 4.1	26.2 ± 4.1	26 ± 4.5	26.1 ± 4.1	26.4 ± 4	25.9 ± 4	26.2 ± 4.7	26.0 ± 4.9	25.9 ± 4.2	26.1 ± 4.4
p-value	n.s.			n.s.			n.s.			n.s.			n.s.		
Waist (cm)	87.17 ± 11.70	88.92 ± 13.6	88.91 ± 13.37	87.80 ± 13.1	88.22 ± 12.8	89.18 ± 13.3	87.20 ± 11.8	88.64 ± 13.2	88.76 ± 13.2	87.78 ± 12.6	88.60 ± 12.8	88.20 ± 13.2	88.12 ± 14.9	88.46 ± 12.9	88.33 ± 12.8
p-value	n.s.			**"0.001**			**"0.004**			n.s.			n.s.		
Hip (cm)	101.6 ± 7.3	103.6 ± 9.2	102.4 ± 7.4	102.61 ± 9.1	106.22 ± 55.8	102.77 ± 7.4	102.37 ± 7.8	106.39 ± 56.4	102.39 ± 7.4	104.14 ± 7.5	102.17 ± 7	105.99 ± 53.0	102.35 ± 9.4	106.67 ± 62.3	102.82 ± 7.9
p-value	n.s.			n.s.			n.s.			n.s.			n.s.		
WHR	0.86 ± 0.09	0.86 ± 0.09	0.87 ± 0.1	0.85 ± 0.1	0.85 ± 0.1	0.87 ± 0.1	0.85 ± 0.1	0.86 ± 0.1	0.86 ± 0.1	0.84 ± 0.1	0.86 ± 0.1	0.85 ± 0.1	0.86 ± 0.1	0.86 ± 0.1	0.86 ± 0.1
p-value	n.s.			n.s.			n.s.			n.s.			n.s.		
**Association analysis with eating behavior factors**															
Disinhibition	4.95 ± 2.88	4.53 ± 3.16	3.81 ± 2.7	4.9 ± 3.11	4.3 ± 3.02	4.04 ± 2.92	4.93 ± 3.16	4.33 ± 2.95	3.94 ± 2.89	4.65 ± 3.04	4.25 ± 2.99	4.41 ± 3.03	4.29 ± 2.92	4.29 ± 3.12	4.43 ± 2.94
p-value	**°0.003**			**°0.032 "0.037**			**°0.006 #0.027 "0.022**			n.s.			n.s.		
Hunger	4.09 ± 2.94	4.04 ± 2.84	3.66 ± 2.66	4.26 ± 2.86	3.83 ± 2.86	3.92 ± 3.10	4.17 ± 2.88	3.85 ± 2.85	3.95 ± 3.04	3.99 ± 3.16	3.99 ± 2.81	3.93 ± 2.98	3.84 ± 2.8	4.07 ± 3.05	3.88 ± 2.86
p-value	n.s.			n.s.			n.s.			n.s.			n.s.		
Restraint	8.79 ± 5.32	7.86 ± 4.73	7.62 ± 4.92	8.52 ± 5.13	7.71 ± 4.67	7.65 ± 5.05	8.52 ± 5.09	7.82 ± 4.77	7.6 ± 4.9	7.42 ± 5.03	7.53 ± 4.88	8.31 ± 4.83	7.8 ± 4.37	7.57 ± 4.88	8.24 ± 4.96
p-value	n.s.			n.s.			n.s.			**°0.048**			n.s.		
**Association analysis with consumer goods**															
Smoking	10.04 ± 8.79	10.07 ± 8.13	9.45 ± 8.09	9.91 ± 8.9	10.34 ± 8.18	8.4 ± 7.4	10.6 ± 9.31	9.58 ± 7.95	10.02 ± 8.48	4.67 ± 3.33	10.32 ± 8.98	9.89 ± 8.23	11.94 ± 8.44	9.39 ± 8.35	9.89 ± 8.52
p-value	n.s.			**#0.044**			n.s.			n.s.			n.s.		
Alcohol	2.83 ± 1.2	2.78 ± 1.17	2.82 ± 1.2	2.88 ± 1.18	2.76 ± 1.11	2.79 ± 1.28	2.84 ± 1.23	2.77 ± 1.12	2.79 ± 1.23	2.84 ± 1.30	2.8 ± 1.22	2.77 ± 1.14	2.9 ± 1.19	2.82 ± 1.19	2.75 ± 1.17
p-value	n.s.			n.s.			n.s.			n.s.			n.s.		
Coffee	1.79 ± 0.72	1.79 ± 0.76	1.74 ± 0.75	1.81 ± 0.76	1.89 ± 0.82	1.75 ± 0.75	1.81 ± 0.73	1.89 ± 0.82	1.71 ± 0.75	1.62 ± 0.68	1.84 ± 0.82	1.81 ± 0.76	1.71 ± 0.61	1.84 ± 0.79	1.80 ± 0.80
p-value	n.s.			n.s.			**#0.043**			n.s.			n.s.		

Data are presented as mean ± SD. Type 2 diabetics were excluded. *P*-values were calculated using additive, dominant (mm + mM vs. MM) and recessive (mm vs mM + MM) model of inheritance using linear regression adjusted for age, gender and lnBMI (except for BMI). Analyses for smoking, alcohol and coffee consumption were adjusted for age and gender; ° = additive model of inheritance; # = dominant model of inheritance; “=recessive model of inheritance; n.s. non-significant; significant *P*-values are highlighted in bold; *N* = number of individuals; BMI = Body Mass Index; WHR = Waist-Hip-Ratio.

Furthermore, we observed decreased waist circumference in homozygous minor allele carriers when applying recessive inheritance models, respectively (rs782881 *P* = 0.001; rs1834150 *P* = 0.004; Table 2). A slightly increased coffee consumption was detected in homozygous or heterozygous minor allele carriers of rs1834150 (*P* = 0.043; Table 2). Further, while carriers of the minor C-allele of rs782881 showed higher cigarette consume per day (dominant model, *P* = 0.044; Table 2), we observed no further relationship to the time period of smoking.

Additional analyses were performed to test the hypothesis that variants within *AKR1B10* are related to glucose, insulin and lipid metabolism. Beside a few nominal associations of rs10232478, rs1834150, rs3778828 and rs4732036 with fasting plasma insulin values, 120 min plasma insulin values, triglycerides levels and 120 min plasma glucose levels (respectively), we did not detect evidence for any significant associations (Additional file 1: Table S1). None of the here reported *P*-values withstand correction for multiple testing (Bonferroni).

### Association analysis in the replication cohort

In order to verify our results we performed linear regression analyses for rs10232478; rs782881 and rs1834150 in an independent German cohort (*N* = 350). Despite failing to reach statistical significance our replication analyses revealed similar trends for higher disinhibition scores in homozygous or heterozygous minor allele carriers (Table 3). We observed a similar effect direction as compared to the Sorbs at rs1834150 and a meta-analysis for rs1834150 resulted in a combined *P* = 0.0096 (Table 4).

**Table 3:** **Association analysis of** ***AKR1B10*** **variants in the replication cohort**

***AKR1B10*** **genotype**									
	**rs10232478**			**rs782881**			**rs1834150**		
**Genotype (** ***N*** **)**	**CC (50)**	**CT (131)**	**TT (99)**	**CC (65)**	**AC (146)**	**AA (81)**	**TT (56)**	**TA (136)**	**AA (82)**
age	27.14 ± 5.46	26.40 ± 4.87	26.53 ± 4.71	26.80 ± 5.54	26.64 ± 4.97	26.91 ± 4.84	26.70 ± 4.82	26.43 ± 5.03	27.50 ± 5.01
p-value	n.s.			n.s.			n.s.		
BMI	27.6 ± 6.53	26.92 ± 6.16	27.21 ± 6.49	27.64 ± 6.67	26.90 ± 5.93	27.36 ± 6.59	26.98 ± 5.94	27.34 ± 6.20	27.11 ± 6.59
p-value	n.s.			n.s.			n.s.		
**association analysis with eating behavior factors**									
Disinhibition	6.64 ± 3.34	6.05 ± 3.12	6.33 ± 3.02	6.15 ± 3.11	6.33 ± 3.22	6.01 ± 3.29	6.29 ± 3.06	6.56 ± 3.31	6.01 ± 3.21
p-value	n.s.			n.s.			n.s.		
Hunger	4.63 ± 2.87	5.57 ± 3.48	5.88 ± 3.26	4.80 ± 3.3	5.72 ± 3.51	5.76 ± 3.42	4.72 ± 3.18	5.99 ± 3.57	5.54 ± 3.37
p-value	**°0.038 "0.036**			n.s.			n.s.		
Restraint	7.85 ± 5.23	6.30 ± 4.41	5.55 ± 4.23	7.27 ± 4.91	6.16 ± 4.22	5.42 ± 4.29	7.35 ± 5.14	5.99 ± 4.13	5.25 ± 4.06
p-value	**°0.010 "0.015**			**°0.027**			**°0.007 #0.049 "0.015**		

Data are presented as mean ± SD. Type 2 diabetics were excluded. *P*-values were calculated using additive, dominant (mm + mM vs. MM) and recessive (mm vs mM + MM) model of inheritance using linear regression adjusted for age, sex and lnBMI (except for BMI); ° = additive model of inheritance; # = dominant model of inheritance; “=recessive model of inheritance; n.s. non-significant; significant *P*-values are highlighted in bold; *N* = number of individuals; BMI = Body Mass Index.

**Table 4:** **Meta-analysis for association with eating behavior factors**

		**Sorbs**			**German cohort**			**Combined**		
**SNP**	**Minor allele**	***P*** **-value**	***β***	**S.E.**	***P*** **-value**	***β***	**S.E.**	***P-value***	**Z-score**	**Direction**
		**Disinhibition**								
rs10232478	C	**0.003**	+0.071	0.024	0.948	−0.016	0.248	0.0194	+2.336	+−
rs782881	C	0.032	+0.357	0.167	0.977	−0.007	0.243	0.0888	+1.702	+−
rs1834150	T	**0.006**	+0.439	0.160	0.544	+0.153	0.252	**0.0096**	+2.591	++
		**Restraint**								
rs10232478	C	0.334	+0.040	0.042	**0.010**	+0.993	0.385	0.0211	+2.306	++
rs782881	C	0.185	+0.381	0.287	0.027	+0.816	0.367	0.0174	+2.378	++
rs1834150	T	0.166	+0.382	0.276	**0.007**	+1.006	0.372	**0.0072**	+2.684	++
		**Hunger**								
rs10232478	C	0.209	+0.030	0.024	0.038	−0.589	0.282	0.818	−0.230	+−
rs782881	C	0.542	+0.106	0.174	0.092	−0.487	0.288	0.6081	−0.513	+−
rs1834150	T	0.785	+0.046	0.168	0.237	−0.357	0.301	0.6459	−0.459	+−

*P*-values were calculated based on effect sizes from linear regression model using additive inheritance model. Significant *P*-values withstanding correction for multiple testing are presented in bold. All data are adjusted for age, gender and lnBMI. All analyses were standardized to the minor allele.

Interestingly, when analyzing a potential correlation of the *AKR1B10* SNPs with restraint scores we observed a nominally significant relationship with increased restraint at the same intragenic variants (rs1834150; rs782881) and the 5′ UTR variant rs10232478 (all *P* ≤ 0.05; Table 3). In a meta-analysis including the Sorbs and the replication cohort we confirmed a nominally significant association of rs1834150 (*P* = 0.00728, Table 4) with restraint. Moreover, we observed a nominal association of rs10232478 in the 5′ untranslated region with the eating behavior factor hunger (*P ≤* 0.05; Table 3).

## Discussion

The human *AKR1B10* was shown to be associated with several types of cancers including lung cancer [10] and liver cancer [9]. *AKR1B10* is highly expressed in the gastrointestinal tract [1] and its expression is triggered via toxic aldehydes like acrolein and HNE present in many daily nutrients [3,13] or through cigarette smoke [10,12]. The reductase AKR1B10 is highly reactive towards this components and may lead to their detoxification during digestion [14]. Interestingly, Higueras-Matas and colleagues found an inhibition of *akr1b10* gene expression in rats more sensitive to drugs like cocaine and ethanol in brain regions known to regulate eating behavior [15]. While a potential role of *AKR1B10* in eating behavior seems plausible in the light of these studies, its role in BMI, waist and hip circumference or the influence on metabolic parameters was never analyzed so far. In the present study we therefore hypothesized *AKR1B10* to play a role in human eating behavior and tested for a relationship to metabolic and anthropometric variables. Using linear regression models we observed a relationship of genetic variants within *AKR1B10* and disinhibition scores in the Sorbs along with similar directions in the replication cohort. Disinhibition is a measure of the loss of cognitive control during eating due to situational factors [40]. This may result in overeating and a higher caloric intake potentially contributing to an increase in body weight [41]. Most of the studies suggest a positive association between high disinhibition levels, BMI and weight gain [42,43]. In contrast, we did not identify any relationship to BMI but observed indications for lower waist circumference at three SNPs which are related to disinhibition. This finding may be explained by the fact that restraint scores at rs1834150, rs782881 and rs10232478 are increased as well. Restraint eaters tend to rigorously control their body weight by permanently controlling their eating habits. These individuals often count calories in order to regulate the amount of food intake [40]. Since both, restraint and disinhibition, are not completely independent, the observed increased restraint scores could probably serve as a counteracting behavior against the disinhibition effect [40,44]. One may argue that high restraint scores were also found to be associated with a higher BMI [45]. However, it needs to be mentioned that restraint is not a homogeneous measure but can be divided in two subscales - rigid control (‘all or nothing’) and flexible control (‘more or less’) [45,46]. While rigid control is associated with higher BMI and eating disorders, flexible restraint is related to decreased BMI and weight loss [43,46]. Furthermore, it was demonstrated that individuals presenting rigid control show increased restraint scores and simultaneously high disinhibition scores which results in increased weight or BMI. In contrast, flexible cognitive control was associated with decreased BMI and weight [41,43,47]. We did not have the opportunity to analyze restraint subscales, but nevertheless our data indicate a counteracting mechanism between disinhibition and restraint which results in a decrease of waist circumference, probably due to a highly flexible cognitive control. Moreover, we found rs1834150 and rs782881 related to intake of consumer goods in the Sorbs. In particular, we show a nominal association at rs1834150 and higher coffee consumption per day and found rs782881 correlated with an increased amount of cigarettes per day. These findings, together with our results from the eating behavior questionnaire FEV, suggest a potential role of *AKR1B10* in human eating behavior. However, it needs to be acknowledged that none of the here observed relationships withstand a Bonferroni correction for multiple testing, thus underscoring the possibility of false positive findings. Therefore, although our data may indicate a role of *AKR1B10* in eating behavior, intake of consumer goods and waist circumference, they need to be interpreted with caution.

Further, we tested for a potential relationship of the *AKR1B10* genetic variants to various variables of the glucose and insulin metabolism as well as lipid profiles. Beside several, rather occasional, non-significant correlations we did not identify any consistent evidence for significant associations with the tested SNPs.

Despite some interesting results, our study is limited at several aspects. (i) Although we present a second independent replication cohort the sample size of our study is rather small which may lead to false positive and false negative results. (ii) There is a large discrepancy in the male/female ratio and in age between the study cohorts which may have, in terms of different eating behavior patterns between men and women, influenced our results as demonstrated by others [48-50]. However, we used gender as covariate in our analysis whereby the influence of discrepancy in the male/female ratio on the results was taken into account. (iii) Due to the lack of data for metabolic variables and consumer goods in the replication cohort we cannot conduct meta-analysis for both sample sets. (iv) While the German version of the three factor eating questionaire (FEV) is validated as reported by Pudel V & Westenhoefer J [31] with internal reliability of the three scales (between r = 0.75 and 0.87) and similar data on internal reliability observed in the Sorbs (Cronbach’s alpha coefficients: disinhibition = 0.744; restraint = 0.856; hunger = 0.745), questions related to consumer goods were not validated. Therefore, our data on consumer goods need to be interpreted with caution.

In conclusion, our data in regard to eating behavior factors support our hypothesis that *AKR1B10* may be a potential contributor to human eating behavior. However, our data need to be cautiously interpreted and larger studies are definitely warranted to replicate and verify the observed effects.

## Conclusions

To the best of our knowledge this is the first evidence for an association of *AKR1B10* with eating behavior. The established role of AKR1B10 in cancers via influencing important signaling pathways like the retinoid acid metabolism [6,8] together with the here presented potential role in human eating behavior may give rise to further scenarios through which AKR1B10 may function in other pathways.

## Additional file

Additional file 1: Table S1. Association analysis of *AKR1B10* variants in the Sorbs population.

## Abbreviations

*AKR1B10*: Human *Aldoketoreductase 1B10* gene
mRNA: Messenger ribonucleic acid
5′ UTR: 5′ Untranslated region
NADP(H): Nicotinamide adenine dinucleotide phosphate
HNE: Hydroxynonenal
LEW: Lewis rats
F344: Fischer344 rats
BMI: Body mass index
*FTO*: *Fat mass and obesity associated* gene
SNP: Single nucleotide polymorphism
*GAD2*: *Glutamate decarboxylase* gene
*LEP*: *Leptin* gene
*CCK*: *Cholecystokinin* gene
FEV: German version of the three factor eating questionnaire
T2D: Type 2 diabetes
OGTT: Oral glucose tolerance test
HDL-C: High density lipoprotein cholesterol
LDL-C: Low density lipoprotein cholesterol
TG: Triglycerides
LD: Linkage disequilibrium
MAF: Minor allele frequency
TD PCR: Touch down polymerase chain reaction
DNA: Deoxyribonucleic acid
N: Number
SD: Standard deviation
n.a.: Not available
