# Supplementary material for: Genetic variants in AKR1B10 associate with human eating behavior
Source: BMC Genet. 2015 Mar 25;16:31. doi: 10.1186/s12863-015-0189-9 (PMC4379593; doi:10.1186/s12863-015-0189-9)
Supplement: Additional file 1: Table S1. — Association analysis of AKR1B10 variants in the Sorbs population. [file 12863_2015_189_MOESM1_ESM.docx]

**Supplementary Table 1.** Association analysis of *AKR1B10* variants in the Sorbs population.

| ***AKR1B10* Genotype** | | | | | | | | | | | | | | | | | | | | |
| --- | --- | --- | --- | --- | --- | --- | --- | --- | --- | --- | --- | --- | --- | --- | --- | --- | --- | --- | --- | --- |
|  | rs10232478 | | | | rs782881 | | | | rs1834150 | | | | | rs3778828 | | | rs4732036 | | | |
| Genotype (*N*) | CC (76) | CT (230) | TT (195) | | CC (114) | AC (263) | AA (150) | | TT (115) | TA (257) | | AA (171) | | AA (37) | GA (201) | GG (293) | CC (49) | TC (210) | TT (280) | |
| **association analysis with glucose metabolism** | | | | | | | | | | | | | | | | | | | | |
| Fasting plasma glucose (mmol/l) | 5.28±0.5 | 5.25±0.5 | 5.23±0.5 | | 5.23±0.5 | 5.25±0.5 | 5.24±0.5 | | 5.21±0.5 | 5.26±0.5 | | 5.23±0.5 | | 5.21±0.5 | 5.24±0.4 | 5.24±0.5 | 5.23±0.5 | 5.24±0.5 | 5.24±0.5 | |
| p-value | n.s. | | | | n.s. | | | | n.s. | | | | | n.s. | | | n.s. | | | |
| 30min plasma glucose (mmol/l) | 8.41±1.7 | 8.28±1.7 | 8.46±1.6 | | 8.46±1.6 | 8.26±1.6 | 8.51±1.7 | | 8.31±1.8 | 8.28±1.6 | | 8.52±1.6 | | 8.55±1.7 | 8.40±1.7 | 8.28±1.6 | 8.16±1.5 | 8.41±1.8 | 8.34±1.6 | |
| p-value | n.s. | | | | n.s. | | | | n.s. | | | | | n.s. | | | n.s. | | | |
| 120 min plasma glucose (mmol/l) | 5.41±1.6 | 5.28±1.6 | 5.57±1.7 | | 5.48±1.8 | 5.29±1.6 | 5.53±1.7 | | 5.35±1.6 | 5.3±1.6 | | 5.57±1.8 | | 5.47±1.8 | 5.47±1.6 | 5.35±1.6 | 4.85±1.6 | 5.52±1.6 | 5.39±1.6 | |
| p-value | n.s. | | | | n.s. | | | | n.s. | | | | | n.s. | | | n.s. | | | |
| **association analysis with insulin metabolism** | | | | | | | | | | | | | | | | | | | | |
| Fasting plasma insulin (pmol/l) | 38.16±26 | 36.96±24.4 | 38.91±20.5 | | 38.24±24.1 | 35.67±20.2 | 40.84±25.8 | | 37.11±23.3 | 36.56±21.4 | | 39.82±24.2 | | 40.74±22.9 | 35.64±19.8 | 38.77±24.7 | 42.21±32.5 | 37.72±22.2 | 36.57±20.5 | |
| p-value | **#0.021** | | | | n.s. | | | | n.s. | | | | | n.s. | | | n.s. | | | |
| 30 min plasma insulin (pmol/l) | 280.86±178.7 | 294.02±185.6 | 294.23±158.9 | | 296.12±215.2 | 284.04±149.5 | 294.13±184 | | 284.76±211.8 | 289.04±161 | | 294.34±163.2 | | 296.66±166.1 | 276.96±153.8 | 298.14±188.7 | 319.09±220.4 | 290.44±151.5 | 284.90±180 | |
| p-value | n.s. | | | | n.s. | | | | n.s. | | | | | n.s. | | | n.s. | | | |
| 120 min plasma insulin (pmol/l) | 157.52±142.6 | 164.83±167.3 | 195.03±156.8 | | 167.90±170.9 | 163.70±147.8 | 191.86±162.9 | | 161.67±169.8 | 167.25±153.0 | | 188.21±153.9 | | 201.32±180 | 170.57±143.3 | 170.32±162.9 | 167.99±153.8 | 176.84±166.2 | 164.63±134 | |
| p-value | **°0.011 #0.005** | | | | n.s. | | | | **°0.048** | | | | | n.s. | | | n.s. | | | |
| **association analysis with lipid profiles** | | | | | | | | | | | | | | | | | | | | |
| Total cholesterol (mmol/l) | 5.27±1 | 5.36±1.1 | 5.32±1.1 | | 5.39±1.1 | 5.38±1.1 | 5.29±1.1 | | 5.37±1.0 | 5.38±1.1 | | 5.27±1.1 | | 5.30±1 | 5.34±1.1 | 5.34±1.1 | 5.32±1.0 | 5.35±1.1 | 5.34±1.1 | |
| p-value | n.s. | | | | n.s. | | | | n.s. | | | | |  | | | n.s. | | | |
| HDL-cholesterol (mmol/l) | 1.68±0.4 | 1.71±0.4 | 1.65±0.4 | | 1.65±0.4 | 1.72±0.4 | 1.67±0.4 | | 1.67±0.4 | 1.72±0.4 | | 1.67±0.4 | | 1.75±0.4 | 1.70±0.4 | 1.67±0.4 | 1.75±0.5 | 1.70±0.4 | 1.67±0.4 | |
| p-value | n.s. | | | | n.s. | | | | n.s. | | | | | n.s. | | | n.s. | | | |
| LDL-cholesterol (mmol/l) | 3.26±0.8 | 3.41±1.0 | 3.39±1.0 | | 3.39±1 | 3.41±1 | 3.32±1 | | 3.36±0.9 | 3.42±1 | | 3.32±1.0 | | 3.4±1.1 | 3.41±0.9 | 3.33±1.0 | 3.21±1.0 | 3.36±1.0 | 3.41±1 | |
| p-value | n.s. | | | | n.s. | | | | n.s. | | | | | n.s. | | | n.s. | | | |
| Triglycerides (mmol/l) | 1.28±1.0 | 1.2±0.8 | | 1.23±0.8 | 1.34±1 | 1.17±0.8 | | 1.26±0.8 | 1.3±1 | | 1.17±0.8 | | 1.25±0.8 | 1.12±0.5 | 1.11±0.6 | 1.32±1 | 1.41±1.1 | 1.23±1 | | 1.19±0.7 |
| p-value | n.s. | | | | n.s. | | | | n.s. | | | | | **°0.019 "0.006** | | | n.s. | | | |

**Supplementary Table 1:** Data are presented as mean ± SD. Type 2 diabetics were excluded. *P*-values were calculated using additive, dominant (mm+mM vs. MM) and recessive (mm vs mM +MM) model of inheritance using linear regression adjusted for age, gender and lnBMI (except for BMI). °=additive model of inheritance; #=dominant model of inheritance; “=recessive model of inheritance; n.s. non-significant; significant *p*-values are highlighted in bold; *N*=number of individuals.
